# Pediatric Gaucher Disease Presenting with Massive Splenomegaly and Hepatic Gaucheroma

**DOI:** 10.3390/children10050869

**Published:** 2023-05-12

**Authors:** Gianluca Bossù, Laura Pedretti, Lorenzo Bertolini, Susanna Esposito

**Affiliations:** 1 Pediatric Clinic, Department of Medicine and Surgery, University Hospital of Parma, 43126 Parma, Italy; gianluca.bossu@unipr.it (G.B.); laurapedre@hotmail.it (L.P.); 2 Unit of Radiologic Sciences, Department of Medicine and Surgery, University Hospital of Parma, 43126 Parma, Italy; lobertolini@ao.pr.it

**Keywords:** Gaucher disease, gaucheroma, enzyme replacement therapy, hepatomegaly, splenomegaly

## Abstract

Gaucher Disease (GD) is a condition resulting from an autosomal recessive inheritance pattern, characterized by a deficiency of the lysosomal enzyme beta-glucocerebrosidase. This leads to the accumulation of glucocerebroside and other glycolipids in multiple tissues, causing damage to various organ systems. The diagnosis of GD can be challenging due to its heterogeneity, non-specific symptoms, and variability across different geographic regions and age groups. Although GD is suspected based on symptoms or signs, the diagnosis is confirmed through the measurement of deficient b-glucocerebrosidase activity and the identification of biallelic pathogenic variants in the *GBA* gene. Intravenous enzyme replacement therapy (ERT) is recommended for GD patients. In this paper, we report a case of a 2-year and 8-month-old girl with massive splenomegaly and radiological finding of hepatic gaucheroma, in whom a genetic study showed homozygous mutation on the *GBA* gene at c.1448T>C (p.Leu483Pro) and certified the diagnosis of GD. This patient represents the youngest child reported to have gaucheroma and also the first one presenting with gaucheroma at the diagnosis and not during the follow up, highlighting that GD should be routinely included in the differential diagnosis of children presenting with splenomegaly and hepatomegaly, taking into account that the early start of ERT can change the natural history of the disease-preventing serious complications.

## 1. Background

Gaucher Disease (GD) is a condition resulting from an autosomal recessive inheritance pattern, characterized by a deficiency of the lysosomal enzyme beta-glucocerebrosidase. This leads to the accumulation of glucocerebroside and other glycolipids in multiple tissues, causing damage to various organ systems [1,2,3]. The genetic basis of GD involves biallelic pathogenic variants in the *GBA* gene, with more than 400 variants identified to date. The incidence of GD at birth varies in different regions of the world, ranging from approximately 0.39 to 5.80 per 100,000 individuals [1].

The diagnosis of GD can be challenging due to its heterogeneity, non-specific symptoms, and variability across different geographic regions and age groups [1]. GD is divided into three main phenotypes: type 1 (chronic, non-neuronopathic), type 2 (acute neuronopathic), and type 3 (chronic neuronopathic) [1]. Children with GD1 commonly exhibit symptoms such as splenomegaly, hepatomegaly, anemia, thrombocytopenia, epistaxis, bruising, delayed growth, and delayed puberty, as well as acute and chronic pain associated with bone disorders. Although GD1 is usually presented in childhood, diagnosis can be delayed until adulthood. Type 2 GD is characterized by early neurological involvement leading to fatality, while type 3 GD has slower progressive neurological involvement. GD3 exhibits the visceral manifestations described in GD1, although they tend to be more severe. Over time, GD3 patients develop neurological symptoms, such as cognitive impairment, myoclonic seizures, ataxia, spasticity, difficulty initiating horizontal eye movements, incomplete vertical eye movements, abnormally slow tracking of objects, and convergence problems with eye squinting and muscle weakness [1,2].

In the majority of cases, oculomotor neurological implication, usually associated with visceral manifestations, occurs before the age of 20. As GD1, GD3 phenotypes are heterogeneous, especially concerning neurological implications. Furthermore, neurological signs may appear several years after the visceral symptoms, even in patients originally diagnosed and treated as affected by GD1 [3].

Patients affected by GD1 have a life expectancy that is usually around ten years shorter than that of the general population, even though advanced therapies could affect the survival of these patients. On the other hand, patients with GD2 usually do not survive beyond two years of age. Children affected by GD3 experience a slower progression of the disease and may live into adulthood. Often GD is not diagnosed or is diagnosed late; this leads to complications and the persistence of symptoms [1]. Although GD is suspected based on symptoms or signs, the diagnosis is confirmed through the measurement of deficient b-glucocerebrosidase activity and the identification of biallelic pathogenic variants in the *GBA* gene [1].

In pediatric patients with symptomatic GD1 and GD3, intravenous enzyme replacement therapy (ERT) is recommended. Although ERT does not slow down or stop the progression of a neurological condition, it has been used off-label for palliative care in some cases of GD2 [4,5]. An oral alternative, substrate reduction therapy (SRT), is available for adult patients with GD1. Additionally, symptomatic therapy may also be provided.

Finally, next-generation SRT, gene therapy, pharmacological chaperone therapy, and histone deacetylase inhibitors represent potential new treatments in development [4,5].

In this paper, we report a case of GD diagnosed in a child with massive splenomegaly and a radiological finding of hepatic gaucheroma, highlighting that GD should be routinely included in the differential diagnosis of children presenting with splenomegaly and hepatomegaly because the early start of ERT can change the natural history of the disease-preventing serious complications.

## 2. Case Report

A 2-year and 8-month-old girl was brought to our attention by her primary care pediatrician. The parents reported persistent abdominal distension during the last 18 months; a series of blood exams performed during the previous year showed anemia, thrombocytopenia, adequate hepato-renal function, and normal hemoglobin chain analysis. At physical examination, significant abdominal distension underlying massive splenomegaly (the splenic pole reached the low hypogastric region) and hepatomegaly was noted without any other significant symptom: general clinical conditions and neurological examination were in order. Development and growth also appeared adequate. Subsequently, the patient was admitted to our Pediatric Clinic Unit for further examination.

Blood exams confirmed anemia and thrombocytopenia (Hb 9.4 g/dL, PLT 62,000/µL) with normal white cell count; blood smear showed anysopoikilocytosis without immature forms. Immunoglobulins and immunophenotype on peripheral blood were in order. Infective diseases (i.e., hepatotropic viruses, leishmania, salmonellae, brucellae, and borrelia) were ruled out; an auto-immune panel comprehensive of ANA, LKM1-2, ASMA, and AMA was carried out and resulted normal for age.

**Figure 1:**
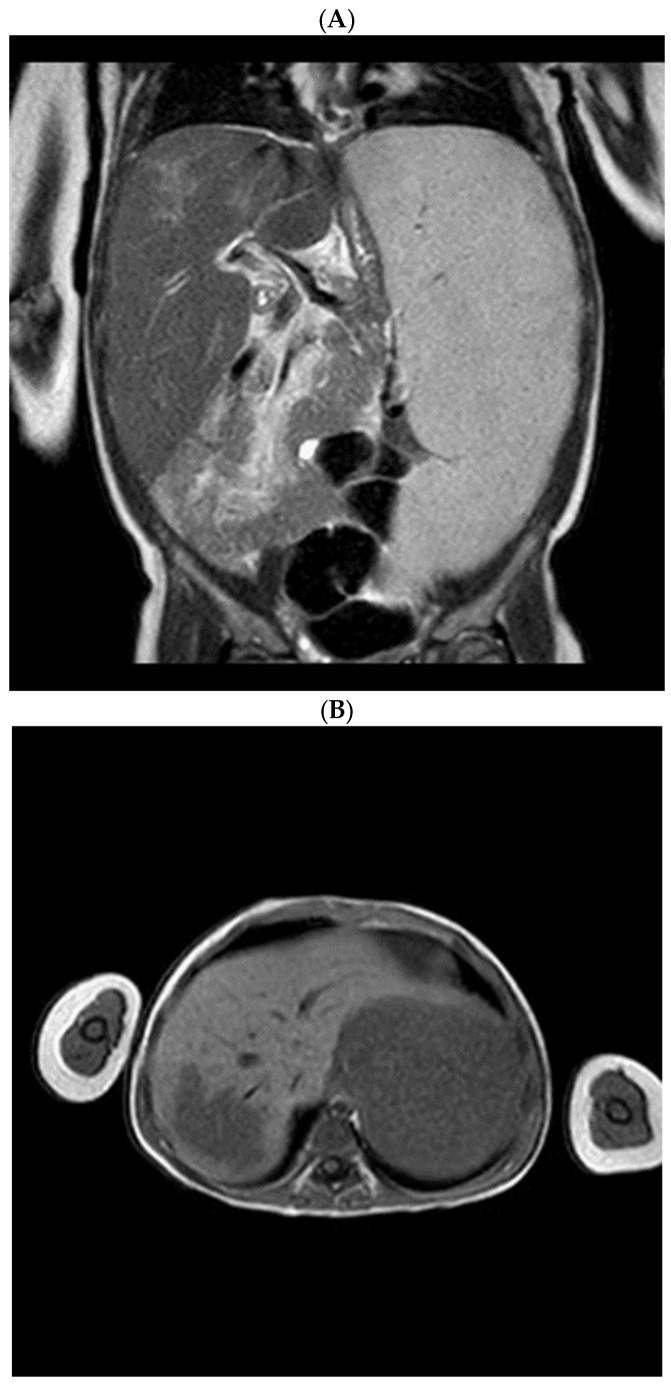
Magnetic resonance imaging shows massive splenomegaly and hepatomegaly (**A**), with a focal hepatic lesion characterized as gaucheroma (**B**).

Radiological assessment of the visceromegaly was performed: computed tomography (CT) and magnetic resonance imaging (MRI) described the massive splenomegaly and highlighted a hepatic focal lesion (Figure 1).

At this point, a dried blood spot (DBS) test for GD and Nieman Pick A/B disease was carried out, revealing decreased activity of β-glucocerebrosidase A; genetic study was then conducted, which confirmed a homozygous mutation in the *GBA* gene at c.1448T>C (p.Leu483Pro), and quantitative measurement of lyso-Gb1 was performed, which showed an elevated concentration (732.4 ng/mL). Therefore, the diagnosis of GD was made.

The case was discussed with pediatric radiologists and a pediatric surgeon, and since alfa-fetoprotein and βHCG came out negative, liver biopsy was excluded, and the hepatic lesion was characterized as hepatic gaucheroma.

A neurological examination, including an EEG, was performed, which did not reveal any neurological changes, and no gaze palsy was observed.

The patient was admitted to the referral center for GD where she began treatment and continued monitoring of the disease. Before starting the treatment, the enzyme glucocerebrosidase was measured again, and it remained deficient (0.3 microMol/L/h). Additionally, the value of lysoGb1 was confirmed to be elevated (782 ng/mL). At the age of 2 years and 10 months, she started ERT with intravenous imiglucerase (Cerezyme) at a dosage of 60 U/kg/dose every 14 days without showing any local or systemic reaction. A lower limb MRI was carried out, which showed marked signs of spinal cord impairment according to the underlying pathology. An echocardiogram and ECG were performed, with normal results. The ophthalmologic examination did not show any pathological changes.

Regarding laboratory tests, 3 months after the start of treatment hemoglobin values recovered (11.2 g/dL), while platelets remained stable (60 × 10^3^/µL). The CYP2D6 genotype study showed normal activity with the following CYP2D6 genotype 2A/2A, making her a normal metabolizer of CYP2D6 substrates, allowing her to be switched to oral therapy with a substrate inhibitor (Eliglustat-Cerdelga) at the age of 18.

According to the pathology follow-up protocol, blood markers of pathology should be monitored every 6 months [4]. In a few months’ time, a check-up of chitotriosidase, lysoGb1, ferritin, acid phosphatase (first value 46.1 U/L; normal value < 4.7), ACE (first value 155 U/L; normal value 35–114), as well as a metabolic–nutritional routine and a complete abdominal ultrasound will be scheduled.

## 3. Discussion

A child presenting with splenomegaly and hepatomegaly like the one described in this case report represents a challenge for pediatricians. Different pathologies in children can cause an enlarged spleen and liver: infectious agents, hematologic disorders, infiltrative diseases, and auto-immune diseases are the most common [3]. In the differential diagnosis, it is important to include GD because, owing to its clinical heterogeneity, there is often a delay in the diagnosis that can lead to severe complications. This child came to our attention several months after the onset of the first symptoms (anemia, thrombocytopenia, and splenomegaly).

To diagnose GD in our patient, DBS was performed to evaluate the enzymatic activity of β-glucocerebrosidase (GCase), followed by an analysis of the *GBA* gene. Supporting the diagnosis, an increase in the lyso-Gb1 biomarker was identified. For a long time, the standard method for diagnosing GD has been to determine the reduced activity of β-glucocerebrosidase (GCase) in peripheral blood cells and to analyze *GBA1* mutations. DBS samples have several advantages, including ease of collection, minimal blood requirement, and straightforward transportation. Nevertheless, DBS has limitations when it comes to measuring GCase activity. Bone-marrow aspiration and biopsies to identify Gaucher cells are no longer considered diagnostic tools for GD and should only be done when assessing another hematologic comorbidity [6]. Additionally, distinguishing Gaucher cells from similar cells observed in hematological diseases or infectious diseases (e.g., chronic myeloid leukemia, atypical mycobacteria, chronic myeloid leukemia, or myelodysplasia, etc.) may be difficult [3]. Given this, we did not perform bone marrow aspiration or biopsies because we were confident in the diagnosis of GD, as there were no warning signs suggesting malignant or hematologic comorbidity.

Regarding plasma biomarkers, they have been utilized for a significant period of time to diagnose and monitor patients with GD. However, traditional biomarkers, such as ACE, ferritin, alkaline phosphatase, and high-density lipoprotein, are not unique to GD, and more specific biomarkers, such as chitotriosidase and CCL18, have limited usefulness. On the other hand, Glucosylsphingosine (lyso-Gb1), the deacylated form of glucocerebroside, has been identified as a potential biomarker with high sensitivity and specificity for the diagnosis of GD [6].

Pathological macrophages in GD release chitotriosidase. While higher plasma chitotriosidase activity is typically observed in type 1 GD patients compared to those with types 2 and 3, it is important to note that increased enzyme activity is not exclusive to GD. In fact, modest elevations in chitotriosidase activity can also be found in various lysosomal and non-lysosomal diseases (i.e., Niemann–Pick disease type C, sarcoidosis, arthritis, multiple sclerosis, thalassemia, and malaria ext.). CCL18 is a member of the C-C chemokine family, and like chitotriosidase, it accumulates in the alternatively activated macrophages present in Gaucher cells in GD. An increase in CCL18 can also be found in various lysosomal and non-lysosomal diseases, similar to chitotriosidase [7].

Lyso-Gb1 is involved in GD-associated bone pathology and chronic inflammation, making it a relevant biomarker. During GD treatment, lyso-Gb1 levels decline rapidly, like other biomarkers. Plasma levels of lyso-Gb1 are much higher in untreated GD patients than in healthy controls and correlate with hepatomegaly, splenomegaly, and platelet counts. Patients with neuronopathic GD have notably elevated levels of plasma lyso-Gb1 compared to those with non-neuronopathic GD [1]. The reliability of diagnosing GD by measuring the lysoGb1 enzyme in DBS and subsequently performing genetic analysis has been demonstrated in a cross-sectional study. Combining lyso-Gb1 measurement with whole-gene sequencing allowed for a 100% accurate diagnosis of GD. This implies that it could potentially become the new standard for screening patients suspected of having GD and it may also serve as a useful tool for patient evaluation and monitoring [6].

We observed hepatomegaly in addition to splenomegaly, and MRI showed a hepatic focal lesion radiologically defined as gaucheroma. Gaucheroma is an uncommon disorder characterized by the formation of a “pseudotumor” consisting of a group of Gaucher cells, typically found in the liver, spleen, bone, and lymph nodes [8].

The prevalence of splenic and hepatic lesions in GD has been reported to range from 19% to 33% and 6% to 20%, respectively, across all age groups [8]. Gaucheromas grow slowly and can appear in both adults and children. Hepatic gaucheromas should be distinguished from hepatocellular carcinoma or lymphoma. Hypodense lesions of the liver and spleen are the distinctive feature of gaucheroma on imaging [9]. In GD1, it has been reported that splenectomy considerably increases the risk of liver gaucheroma. Although the risk of gaucheroma may be higher in cases where GD diagnosis and treatment are delayed, there is no clear association between the risk of gaucheroma and the severity of GD or the presence of lymphadenopathy or malignant alterations [9]. In patients with GD, routine imaging should be performed to identify gaucheromas. If diagnosed with liver Gaucheroma, patients should be referred to a hepatology specialist and monitored regularly using MRI or CT for adults and ultrasound for pediatric patients. For spleen gaucheroma, patients should be re-evaluated after one year and then monitored every 2–3 years using ultrasound or MRI [9].

According to our knowledge and the literature available [10,11,12,13], this case represents the youngest patient reported as having gaucheroma and also the first one presenting with gaucheroma at the diagnosis and not during the follow-up. Since a biopsy of gaucheromas may pose a risk of further seeding [9] and could be dangerous in this patient for the presence of massive splenomegaly, after consultation with a pediatric surgeon, we felt confident that liver biopsy could be avoided. Tumoral markers were negative, according to the radiologist, the lesion could be well characterized [11,14], and a genetic study confirmed GD diagnosis.

As previously mentioned, over 400 mutations have been identified in the *GBA1* gene: c.1226A>G (N370S), c.1448T>C (L444P), c.84dup, c.115+1G>A (IVS2+1G>A) and RecNciI are the most common [3]. The association between genotype and phenotype can assist in determining the risk level and clinical approach for individuals with inherited diseases. GBA genotype may be considered a significant contributor to GD phenotype. On the other hand, determining *GBA* gene variants may have a limited value in predicting organ involvement and disease severity. Patients with the same GBA genotype, and even siblings, may have different clinical manifestations. In addition, the GBA genotype does not seem to determine the response to ERT.

Despite these limitations, the GBA genotype can be useful to distinguish between the classic neuronopathic and non-neuronopathic forms [1]. If the c.1226A>G (N370S) mutation is identified in homozygosity or heterozygosity, the risk of neurological involvement (GD2 or GD3) can be excluded. Patients with the N370S mutation may remain asymptomatic for a long time, while in the case of homozygosity for the L444P mutation, there is an increased risk for the development of neurological alterations (GD2 or GD3) [3]. Patients homozygous for the rare c.1342G>C (D409H) mutation are at a higher risk of developing damage to their cardiac valves. Heterozygous or homozygous mutation in the GBA1 gene (i.e., c.1226A>G (N370S), c.1448T>C (L444P) ext.), gives an increased risk of developing Parkinson’s disease [3].

Homozygosity for c.1448T>C (p.Leu483Pro) found in our patient has been usually associated with GD3 [1,12,13], which has usually a slower neurological progression than GD2. Despite characteristic manifestations, there is currently no consensus for the diagnosis of this form other than neurological involvement in a patient with proven GD not explained by other causes [13,14]. Neurological signs may appear years after the onset of visceral manifestations. Indeed, even patients initially diagnosed with GD1 may be affected by GD3 [3].

Since our patient had no neurologic signs, both clinical and instrumental, it was not possible to define if she was affected by GD1 or GD3, and this will presumably become more evident during the monitoring of the disease.

In the diagnostic algorithm of GD, an eventual GD3 definition is of significative importance because ERT does not cross the blood–brain barrier at therapeutic levels, thus potentially having no impact on neurological symptoms and deterioration [15,16,17,18]. Different pharmacological trials regarding novel therapies for GD3 are ongoing, including gene therapy [19] and molecular chaperones [20]; moreover, a trial on the safety and pharmacokinetics of eliglustat (SRT) in pediatric patients with GD1 and GD3 is underway [21] and will hopefully provide newer and more feasible tools to treat these patients [22]. Nevertheless, in pediatric patients with GD1 or GD3 presenting with symptoms, an immediate start of treatment is recommended [4,5]. ERT therapy for moderately affected patients starts at 30 U/kg per 2 weeks; the dosage can be increased if therapeutic goals are not met, and usually, patients with severe symptoms start at 60 U/kg [23]. It is proven that ERT has a rapid positive effect on the most frequent manifestations, such as hepatosplenomegaly, anemia, and thrombocytopenia, thus preventing major complications in the short-term period [20]. In the youngest patients, ERT also favors an adequate development of the bone, improving bone density and reducing osteonecrosis [24,25]. In GD, skeletal damages such as vertebral collapse and fractures are serious complications that can heavily impair children’s development, causing long-term disability.

Although individuals with the p.Leu483Pr genotype often show neurologic manifestations, cases of adults treated with ERT showing no neurological worsening up to the age of 50 years are reported in the literature [26]. These adults were treated with ERT, and it is suspected that ERT reduces the general proinflammatory state seen in GD with an indirect effect on nervous system integrity [1].

Regarding gaucheroma, at the moment there is no clear indication in the literature on treatment regimen [12]. Similar cases to the one reported in our patient have been described in older children and appeared during treatment with ERT [10,11]. Recently, the case of an adult with GD3 presenting a large mesenteric gaucheroma has been described [27]: the patient was already receiving ERT, so SRT was implemented. Follow-up imaging studies after the beginning of combination therapy of SRT and ERT performed after 31 months showed a significant reduction of the mesenteric mass, and the patient reported an improvement in abdominal discomfort. Early start of SRT therapy in childhood might prevent the development of gaucheroma, and combination therapy could be the key to treating this pseudotumor, although data on SRT pediatric pharmacokinetics [21] are awaited to establish appropriate SRT dosage for children.

## 4. Conclusions

This case shows that GD should be routinely included in the differential diagnosis of children presenting with splenomegaly and hepatomegaly, considering that the early start of ERT can change the disease progression, preventing serious complications. There is no clear evidence regarding the efficacy of ERT for gaucheroma, but hopefully, new effective and safe treatments will be available in the next few years.

## Data Availability

All the data are included in the manuscript.
